# Does Tobacco Smoking Affect Vaccine-Induced Immune Response? A Systematic Review and Meta-Analysis

**DOI:** 10.3390/vaccines12111260

**Published:** 2024-11-07

**Authors:** Federica Valeriani, Carmela Protano, Angela Pozzoli, Katia Vitale, Fabrizio Liguori, Giorgio Liguori, Francesca Gallè

**Affiliations:** 1 Department of Movement, Human, and Health Sciences, University of Rome Foro Italico, 00135 Rome, Italy; federica.valeriani@uniroma4.it; 2 Department of Public Health and Infectious Diseases, Sapienza University of Rome, 00161 Rome, Italy; carmela.protano@uniroma1.it (C.P.); angela.pozzoli@uniroma1.it (A.P.); katia.vitale@uniroma1.it (K.V.); 3 Department of Economics and Legal Studies, University of Naples “Parthenope”, Via Generale Parisi 13, 80132 Naples, Italy; fabrizio.liguori@studenti.uniparthenope.it; 4 Department of Medical, Movement and Wellbeing Sciences, University of Naples “Parthenope”, 80133 Naples, Italy; giorgio.liguori@uniparthenope.it

**Keywords:** tobacco smoking, active immunization, antibody titre, systematic review, meta-analysis

## Abstract

**Background**. Causing approximately 8 million deaths each year, tobacco smoking represents a significant public health concern. Evidence shows that smoking significantly impairs antibody production and immune cell activity following vaccination. **Objectives**. This review aims to provide a comprehensive overview of the literature regarding how smoking reduces the effectiveness of active immunization by affecting vaccine-induced immune response. **Methods**. This study was performed according to the PRISMA guidelines, and the protocol was registered on the PROSPERO platform (ID: CRD42024582638). PubMed, Scopus and Web of Science were consulted as bibliographic and citation databases. Studies published in Italian and English and that aimed to investigate the effects of exposure to active and passive tobacco smoking on vaccine-induced immune response were included. **Results**. Thirty-four studies were selected. Overall, a decrease in antibody levels and avidity and in immune cell production were observed in individuals exposed to smoke. The meta-analysis showed a weighted mean difference between smokers and non-smokers equal to 0.65 (95% CI: 0.10–1.19, *p* = 0.02) for vaccinations against COVID-19, influenza, pneumococcus, HBV, HPV, tetanus, pertussis, polio, haemophilus influenzae type b, measles–mumps–rubella, and recurrent urinary tract infections. **Conclusions**. Smoking cessation campaigns should be considered in order to increase the effectiveness of vaccination programs. Furthermore, the opportunity to adopt different vaccine dosing schemes for smokers and non-smokers, especially in acute epidemics, should be considered.

## 1. Introduction

Smoking is one of the most common and harmful habits worldwide and it represents a huge and widespread public health problem [1]. The global tobacco epidemic has severe health and economic impacts, which makes tobacco control an essential public health issue [2]. Despite the plethora of initiatives implemented to combat this habit, in 2020, the use of tobacco products remained prevalent among 22.3% of the global population (36.7% of men and 7.8% of women) [1]. In recent decades, new alternative types of tobacco smoke have also appeared on the market and become available for sale, such as electronic nicotine delivery systems (ENDSs). The most common of these products are electronic cigarettes (e-cigarettes), which have also begun to receive much attention from the scientific community [3]. Other new devices include HTPs, which allow users to inhale nicotine by heating reconstituted tobacco to 350 degrees Celsius, in contrast to the way in which it is inhaled via traditional cigarettes [4]. Together with hypertension, smoking constitutes the main risk worldwide for premature death and disability among all age groups [5]. Tobacco smoking, in fact, kills approximately 8 million people a year worldwide. Of these deaths, almost 7 million are directly related to tobacco consumption and over 1.3 million are caused by second-hand smoke exposure for non-smokers [1]. As a result of exposure to tobacco toxins, smoking has several adverse health effects, including an increased risk of developing lung cancer, chronic obstructive pulmonary disease, cardiovascular disease, respiratory tract infections from bacteria and viruses, and other conditions [6]. Furthermore, smoking affects the immune system [7]. It has been demonstrated that cigarette smoking is associated with an elevated risk of developing a number of immunological disorders. These include systemic inflammatory diseases, such as rheumatoid arthritis, and autoimmune diseases, such as allergies and transplant rejection. Additionally, studies have demonstrated that smoking impairs the immune system’s ability to respond to external antigens, which compromises its capacity to combat infections [8,9,10]. Investigations into the effects of smoking on the immune system have also looked into the humoral response following immunization and the persistence of protection elicited by a number of vaccines. According to certain research, active smokers have a higher chance of having low immunoglobulin G (IgG) avidity or lower levels of vaccine-induced antibodies [7]. This hypothesis, if confirmed, could constitute a future public health problem, as the reduction of vaccine effectiveness would reduce vaccination coverage, with a negative impact on both health and public spending. It is widely recognized that smoking affects the humoral response after immunization. However, evidence is insufficient to draw firm conclusions or consensus on this issue. This is probably because the study population varies depending on factors such as age, comorbidities, and smoking history, or because different types of vaccines have different effects [7]. Given the high prevalence of smoking worldwide and the primary importance of vaccines as a priority public health issue, this review aims to provide a comprehensive overview of the literature on the impact of tobacco smoking on vaccines’ effectiveness. In particular, the research question of the review is as follows: considering all types of vaccines, how does smoking affect the vaccine-induced immune response?

## 2. Materials and Methods

### 2.1. Research Strategy

This review was performed based on the Preferred Reporting Items for Systematic Reviews and Meta-Analyses (PRISMA) statement. This tool reports guidelines that are designed to help researchers to perform and report the reason for the systematic review, the methods used, and the main findings [11]. The protocol was recorded on PROSPERO (ID: CRD42024582638). The bibliographic and citation databases used for the search were Scopus, PubMed (Medline) and Web of Science (Science and Social Science Citation Index). The following keywords, with Boolean operators such as AND–OR, were utilized: (“cigarette smoke” OR “smoke” OR “secondhand smoke” OR “thirdhand smoke” OR “smoker *” OR “active smoking” OR “e-cigarette *”) AND (“vaccine *” OR “vaccination *” OR “vaccine efficacy” OR “vaccination efficacy” OR “immunization” OR “antibody response to vaccination” OR “humoral immunity” OR “vaccine antibody levels”). The search was conducted from 9 August 2024 to 30 August 2024. The research included all of the articles published from inception of each database to 30 August 2024.

### 2.2. Inclusion and Exclusion Criteria

All studies that were published in Italian and English language and which aimed to investigate the effects of exposure to active and/or passive tobacco smoking, including the use of new tobacco products such as e-cigarettes and heat-not-burn products, on vaccine-induced immune response were included. Any observational, semi-experimental and experimental studies on humans were considered, whereas studies not reporting original data, such as reviews, systematic reviews, case studies, proceedings, qualitative investigations, book chapters, editorials, or commentary studies, were excluded. The evaluation of further critical and systematic review and/or meta-analysis references was undertaken with the aim of finding additional published literature. Any article that did not meet the requirements for inclusion was excluded. In order to structure the research question, the PICOS model was used, as follows:

- Population: all people (individuals of all gender, age, ethnicity and health conditions) vaccinated against any vaccine-preventable disease.
- Intervention: active and/or passive tobacco smoking.
- Control: age-, gender- and condition-matched non-smoking control group (if present).
- Outcomes: effects of active and/or passive tobacco smoking on vaccine-induced immune response.
- Study: observational studies, semi-experimental and experimental studies on humans. All studies that did not satisfy the inclusion criteria were excluded.

The references of all the chosen papers were exported on Zotero citation management software (RRID:SCR_013784), for the purpose of removing any duplicates and to assess the relevance of each article. Firstly, by reviewing the titles and abstracts of the potentially eligible studies, three researchers (A.P., K.V. and F.L.) independently verified the information. The process of examining and evaluating was aided by four topic experts: C.P., F.G., F.V., and C.P.P. The full text of each included article was then assessed separately by the two investigators (A.P., K.V.). Any disagreements over the chosen articles were discussed and resolved by the group.

### 2.3. Risk of Bias Assessment

Thirty-four studies, of which four were randomized clinical trials, one was a non-randomized clinical trial, eight were cross-sectional studies and twenty-one were cohort studies, were obtained at the end of the evaluation and selection process. Two investigators (A.P., K.V.) independently estimated the quality of each study through the NOS tools and the checklist to evaluate a report of a non-pharmacological trial (CLEAR NPT). The NOS scale, which was adjusted for the cohort, case-control, and cross-sectional studies assessed, was used to assess the quality of observational research. The overall rating was calculated using this scale. The average of the writers’ ratings was used to establish the final rating of each article. The scale has several numbers of questions and scores for different types of research, as follows: 8 questions with a maximum score of 9 for case-control and cohort studies, and 6 questions with a maximum score of 7 for cross-sectional studies. A score from 7 to 9 indicates good quality, from 5 to 6 indicates average quality, and from 0 to 4 indicates poor quality. The CLEAR NPT checklist was applied specifically to elaborate the quality of non-pharmacological clinical trials. It is composed of 10 questions with 3 answer options each (yes, no, not reported). These questions allowed us to determine the risk of bias for each study (high, medium and low bias risk). Each affirmative answer corresponds to 1 point: 10 to 8 points means a low bias risk, 7 to 5 indicates a medium bias risk and lower than 5 indicates a high bias risk. The group of four researchers debated and solved any dissension about the score obtained for each study. The data extraction table includes the quality assessment. Additionally, first author, publication year, nation, sponsorship, study design, sample size, population characteristics, tobacco smoking type, vaccine type, and the main results of each article are listed in this table.

### 2.4. Data Synthesis

We also performed a comprehensive meta-analysis in order to assess the impact of smoking on vaccine efficacy [12]. Odds ratios were calculated as effect size (ES) estimates. The reported vaccine efficacy (VE) was calculated using the equation [ratio = (1 − VE%)/100]. The same method was used to obtain 95% confidence intervals (CI). The following risk classification was used to interpret the VE: very low vaccine efficacy (VE 0–0.3), low vaccine efficacy (VE 0.4–0.5), slight vaccine efficacy (VE 0.6–0.8), no effect (VE 0.9–1.1), high vaccine efficacy (VE 1.2–1.6), high vaccine efficacy (VE > 1.7). Cochran’s Q (Hedges Q statistic) was employed for the purpose of assessing the diversity of the named studies and for the testing of the classical measure of diversity. The following thresholds were employed in the interpretation of I^2^: a value of less than 25% indicates low heterogeneity, a value of less than 50% indicates moderate heterogeneity, and a value of greater than 75% indicates high heterogeneity. In order to estimate potential publication bias due to the high volume of samples included, the Egger’s test and channel plot were performed. Subgroup analyses were conducted for outcomes reported in studies comprising two or more groups within each subgroup. To identify the anticipated sources of heterogeneity, meta-regression and subgroup analysis were employed [13]. A series of predefined subgroup analyses were conducted, taking into account the type of vaccine, sample size, gender, age range, publication year, and methodological quality of the study and design of study. A meta-regression analysis was conducted using the Comprehensive Meta-Analysis software with the unrestricted maximum likelihood method [13].

## 3. Results

**Figure 1:**
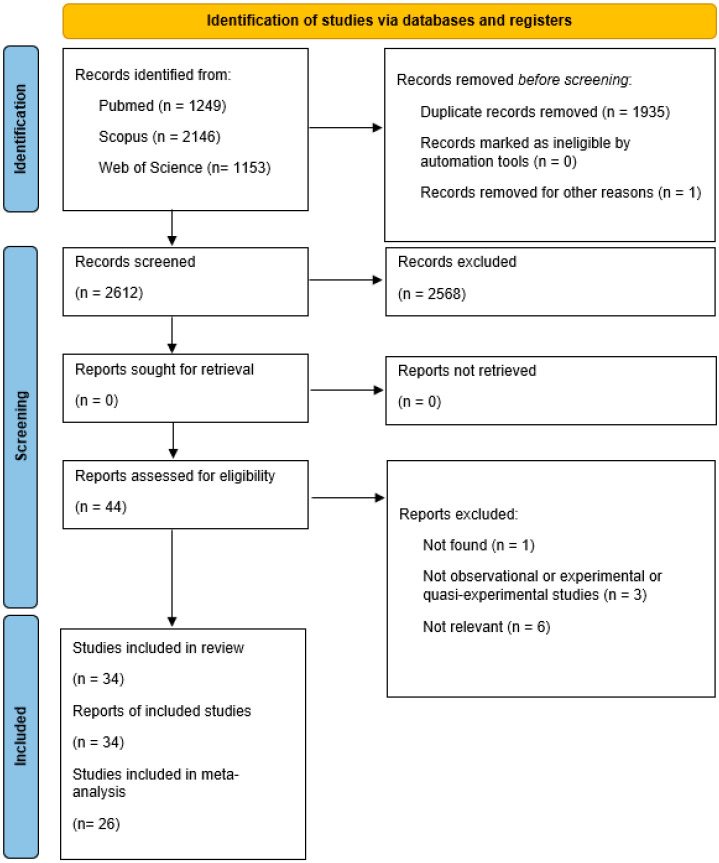
PRISMA 2020 flow diagram for studies included in the analysis.

Using the search terms, 4548 records were found across the databases. After the removal of 1935 duplicates and 1 article that had been withdrawn from publication, 2612 items were screened by title and abstract. Of the 44 records remaining, 10 were eliminated because they did not match the qualifying requirements. The final systematic review comprised 34 publications, with 26 papers included in the meta-analysis. Figure 1 outlines the selection procedure.

Table 1 reports the bibliographic information, study design and country, potential corporate sponsorship, sample characteristics, type of smoking, type of vaccination, main results and quality of the selected studies.

Five of the included studies were clinical trials—four [14,16,17,23] were randomized and one [15] was not randomized. According to the CLEAR NPT checklist for bias risk testing, three clinical trials have a medium bias risk and two a low bias risk. Twenty-one of the selected articles [19,20,21,22,24,26,27,28,29,31,32,33,34,37,38,39,42,43,44,45,47] reported cohort studies and the remaining eight reported cross-sectional studies [18,25,30,35,36,40,41,46]. According to the NOS scale for the quality of cohort and cross-sectional studies, twenty-four of them were good quality, four were shown to be of fair quality and only one was shown to be of poor quality. The studies took place in different continents (Europe, Australia, Asia, USA) and were carried out over the period from 1976 to 2024. Vaccinations against COVID-19, influenza, pneumococcus, HBV, HPV, tetanus, pertussis, polio, haemophilus influenzae type b, measles–mumps–rubella, and recurrent urinary tract infections were analysed.

The study populations were divided into non-smokers and smokers. In this last category, two subgroups were identified according to smoking habits: subjects exposed to passive smoking and subjects exposed to active smoking. Among active smokers, a further differentiation between current and former smokers was made. In addition, attention was drawn to the various means for tobacco smoking, such as cigarettes, pipes, cigars, new electronic nicotine delivery systems (ENDSs) such as electronic cigarettes, and heated tobacco products (HTPs). Regarding the various types of smoking, thirty-three articles focused on active tobacco smoking [14,15,16,17,19,20,21,22,23,24,25,26,27,28,29,30,31,32,33,34,35,36,37,38,39,40,41,42,43,44,45,46,47], but only one of these covered cigar and pipe smoking in addition to cigarette smoking [17]. This article showed a significant negative correlation between smoking and influenza vaccine-induced antibody titre.

No studies related to the use of electronic cigarettes were found, whereas two studies were identified regarding both classic tobacco smoking and heated tobacco products [36,40]. Both of these studies evaluated the effect of smoking on the change in antibody titre induced by the BNT162b2 COVID-19 mRNA vaccine. The first shows a significant negative correlation between the Fagerstrom test and IgG levels in users of both types of tobacco, but a non-significant negative correlation was found between cotinine and antibody levels. In contrast, the second study highlights that exclusive cigarette smokers have a significant reduction in antibody titre, which is non-significant in heated tobacco users.

Significant reduction in antibody titre (pertussis, polio, haemophilus influenzae type b, measles–mumps–rubella vaccines) has also been shown with regard to the exposure to second-hand smoke, but only one study about this issue was included in the systematic review [18].

Reduced antibody titre has been studied as an outcome in several other studies concerning active smoking [15,19,23,26,27,30,31,32,33,38,39,41,42,44,45,46]. In almost all cases, the negative correlation was found to be statistically significant in relation to two vaccinations: different types of COVID-19 vaccine [26,27,30,31,32,33,38,39,42,44,45] and the pneumococcal vaccine [19]. The latter article also highlights the significant association between the number of cigarettes smoked per day and the number of packs smoked per year with reduced antibody levels. The negative correlation between active tobacco smoking and reduced antibody titre was found to be non-statistically significant for anti-HBV [15], tetanus [23], and anti-COVID-19 vaccination [41,46]. Some peculiarities emerged from the analysed studies. The study of Nomura et al. [28] reported sex differences within the smoking category: the median percentage change in antibody titres was significantly lower in female smokers, indicating a more rapid antibody decline in women than in men. On the other hand, the second article of the same group [27] stated that the difference between sexes in age-adjusted median antibody titres in those who had always smoked vs. those who had never smoked had no significant value. Nevertheless, the same study verifies a significant reduction in vaccine-induced antibody titre not only in comparisons between current smokers and those who have never smoked and between former smokers and those who have never smoked, but also between current and ex-smokers. This striking difference between the latter two categories could be of fundamental importance, as it would imply a potential increase in vaccine efficacy in those who decide to quit smoking. The second most investigated outcome was the negative correlation between active tobacco smoking and vaccine failure, which was found to be statistically significant for HBV [16,22,24], tetanus [23], and COVID-19 BNT162b2 mRNA [29] vaccinations. Nevertheless, some studies have shown a negative but not statistically significant correlation, particularly for HBV vaccination [15] and anti-COVID-19 [25]. A limited number of studies have evaluated the correlation between active tobacco smoking and vaccination efficacy as an outcome. All of these showed a negative association, which was statistically significant for the influenza [17], HBV [47] and HPV [35] vaccines. A negative but non-significant correlation emerged for this latter vaccination in the study by Namujju et al. [21], and for MV140 vaccine in that of Ramirez Sevilla et al. [37].

Finally, the negative correlation between active tobacco smoking and antibody longevity for COVID-19 BNT162b2 mRNA [31,34] and influenza [14] vaccines found was found to be statistically significant. Regarding the latter vaccination, only the study by Nath et al. [20] found no association between active tobacco smoking and response to vaccine.

**Figure 2:**
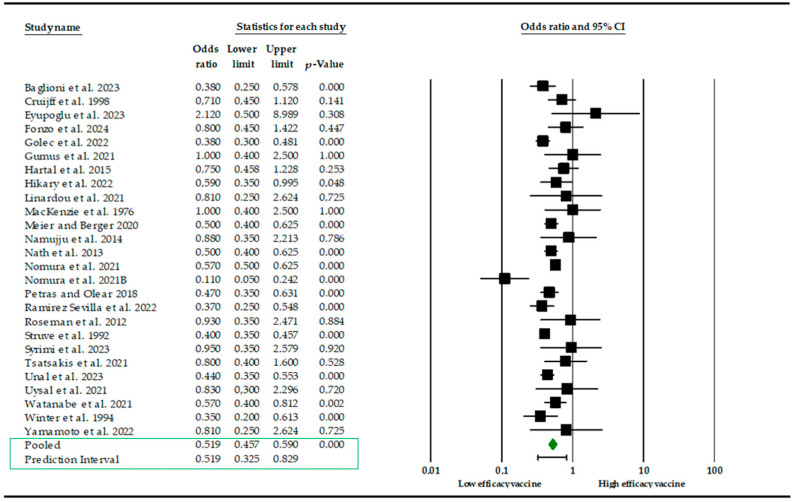
Forest plot of the efficacy of vaccines (VE) after smoking exposure [14,15,16,17,19,20,21,22,23,24,25,26,27,28,30,32,33,34,35,37,40,41,42,43,45,46,47].

The meta-analysis was based on a synthesis of the findings from 25 studies. Eight studies were excluded from the analysis due to the unavailability of the requisite data. The effect size index is represented by the odds ratio as vaccine efficacy (VE). As illustrated in Figure 2, the meta-analysis results indicate that smokers are at an elevated risk of producing a diminished level of immune cells (VE, 0.519; 95% CI, 0.457–0.590, Figure 2).

**Figure 3:**
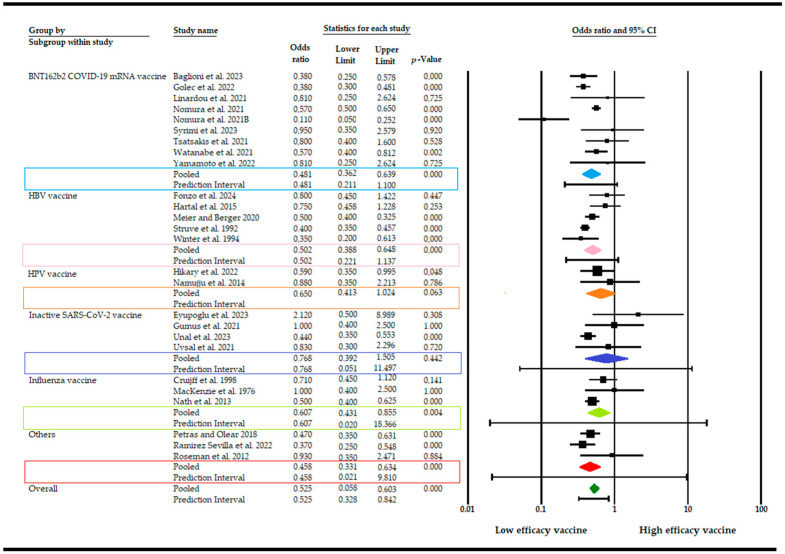
Forest plot of the efficacy of vaccines (VE) after smoking exposure with subgroup analysis [14,15,16,17,19,20,21,22,23,24,25,26,27,28,30,32,33,34,35,37,40,41,42,43,45,46,47].

The effects of the individual studies exhibited a moderate level of heterogeneity (Q = 65.16, d.f. = 25, *p*-value < 0.001, I^2^ = 62%). Studies incorporated for the analysis demonstrated slight heterogeneity (I^2^ = 62%; *p*-value < 0.001), which was adequately addressed with a weighted inverse variance random effects model. For further analysis of this heterogeneity, we used a funnel plot as a subjective assessment and conducted subgroup analysis, sensitivity analysis, and univariate meta-regression for objective assessment of the aetiologies of heterogeneity. Subgroup analysis (Figure 3) based on the type of vaccine revealed that the highest elevated risk of producing a diminished level of immune cells is linked to the BNT162b2 COVID-19 mRNA vaccine and HBV (VE, 0.481; 95% CI, 0.362–0.639; I^2^ = 73.2%; *p* < 0.001; VE, 0.502; 95% CI, 0.388–0.648; I^2^ = 63%; *p* < 0.001), while the lowest was in inactive SARS-CoV-2 vaccine (VE, 1.09; 95% CI, 0.392–1.505; I^2^ = 63%; *p* < 0.001).

**Figure A1:**
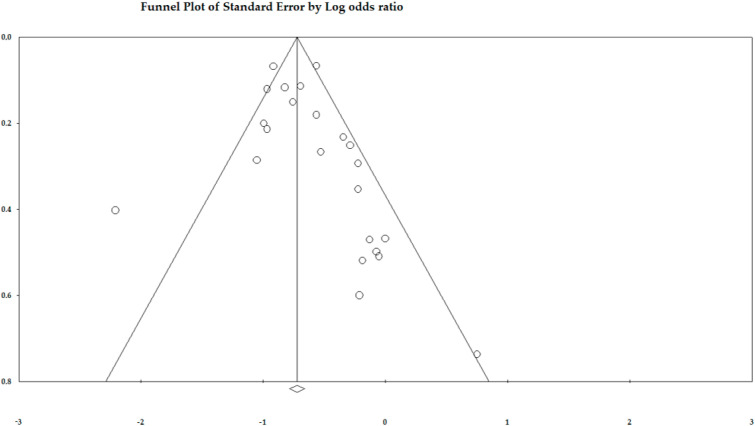
Funnel plots for meta-analysis regarding the efficacy of vaccines (VE) after smoking exposure.

Additionally, subgroup analysis was also based on the design and quality of study. The risk of producing a diminished level of immune cells is similar with reference to the design of the study (VE, 0.514; 95% CI, 0.217–1.739; I^2^ = 64%; *p* < 0.001) and the same is true for quality (VE, 0.509; 95% CI: 0.4069–0.552, I^2^ = 61%; *p* < 0.001). To investigate the factors influencing the efficacy of the vaccine in smokers, a meta-regression analysis was performed, considering sample size, gender, age, study designs and publication year, as well as the type of vaccine. The results demonstrate that the effect was not dependent on the proportion of female participants in the sample (*p* = 0.09) or the mean age of participants (*p* = 0.140). Moreover, the study design (*p* = 0.690) and quality of study (*p* = 0.450) seem not to have influenced the results. Conversely, the efficacy of the vaccine exhibited a modest decline contingent on the specific formulation, indicating that smoke exposure might exert a more pronounced influence on vaccine efficacy relative to other variables (Q = 2.62, d.f. = 3, *p* < 0.05). A comprehensive examination of the impact of smoke exposure on vaccine efficacy was conducted, utilizing data from 26 disparate sources. A sensitivity analysis of the level of immune cells was conducted using a random effects model. The exclusion of studies with a smaller sample size resulted in a slight difference in the pooled VE, which did not significantly affect the stability of the summary result. The potential for publication bias was evaluated both subjectively and objectively. Subjectively, the funnel plot (Figure A1) was examined, and objectively, Egger’s regression test was conducted, yielding a *p*-value of 0.02 and thus indicating that we cannot exclude the absence of small missing studies and, consequently, the absence of publication bias.

## 4. Discussion

The systematic review and meta-analysis were conducted to explore the impact of smoke on vaccine efficacy. The final systematic review comprised 34 publications, with 26 papers included in the meta-analysis. The selected articles suggest that the exposure to tobacco may negatively affect the production of vaccine-induced antibodies after immunization, independently of smoking type.

Evidence shows that smoking affects the immune system and cigarette smoking is linked to an increased risk of developing several immunological diseases, including allergies, transplant rejection and rheumatoid arthritis [7]. It also impairs the immune system’s ability to respond to external antigens, increasing the risk of infection [48]. Smoking harms the immune system in multiple ways, affecting both innate and adaptive immunity [7]. Smoking lowers immune cell counts, although the effect of tobacco chemicals varies depending on individual smoking habits and the cells studied. In particular, analyses of Ig have revealed a decreased production of IgA, IgG, and IgM associated with smoking, affecting the ability to generate memory cells [31,49,50,51,52]. Furthermore, effects of smoking have been observed on inflammatory cytokine and chemokine production, chronic inflammation, and reduced T cell proliferation [51,52,53]. It has been demonstrated that smoking, in addition to latent cytomegalovirus infection and body mass index, constitutes a significant external contributor to the development of the disease, almost as important as age, sex and genetics [54]. It has been established that smoking affects both innate and adaptive immune responses [55,56]. The effect of smoking on innate responses is observed to diminish rapidly following cessation of the habit, while the effect on adaptive responses is observed to persist for a longer period of time. Some findings have revealed that, subsequent to smoking cessation, cytokine secretion in the innate immune response reverts to the levels observed in non-smokers but that the effects on the adaptive response persist for years, probably due the epigenetic memory [57,58,59]. In particular, Saint-André et al. have demonstrated that smoking exerts an influence on cytokine responses, the methylation of signal trans-activators and on the regulators of metabolism [60]. Moreover, it seems that several types of smoking, including e-cigarette aerosols, can variously affect the immune system [61,62]. Indeed, although e-cigarettes were first introduced in 2007 as a mean of assisting smokers to quit, specific modifications to the inflammatory and immune milieu associated with long-term use have been identified, probably due to the formation of new decomposition compounds of questionable toxicity [63].

This systematic review has highlighted that smoking detrimentally affects the vaccine-induced immune response. In fact, nearly all of the selected articles showed a negative relationship between tobacco exposure and response to vaccines, whether expressed as antibody titres or vaccine effectiveness, vaccine failure or antibody longevity.

Our meta-analysis results suggest that the efficacy of the vaccine may be reduced by exposure to tobacco, depending on the specific typology of vaccines. The observed heterogeneity among the studies (I^2^ = 62%; *p*-value < 0.001) necessitated additional analyses to elucidate the underlying causes. Although a weighted inverse variance random effects model was inadequate for addressing the observed heterogeneity, a combination of subjective assessments through funnel plots and objective analyses, including subgroup analysis, sensitivity analysis, and univariate meta-regression, was employed to gain further insight. The aforementioned approaches were employed to elucidate the potential sources of heterogeneity, thereby affording a more nuanced comprehension of the variability in vaccine efficacy (VE) reported. Subgroup analysis based on the type of vaccine revealed that the COVID-19 mRNA, HBV, MV140, and tetanus vaccines exhibited a greater reduction in efficacy following smoking. Moreover, the efficacy of the vaccine may be influenced by the route of administration and the smoker’s habits. It has been demonstrated that cigarette smoke can lead to a reduction in the responsiveness of the immune system to mucosal vaccines, as evidenced by a decline in antibody production and an increase in the incidence of adverse reactions [64]. Although the results have yet to be corroborated by subsequent studies, it appears that smoking does not impede antibody production in response to the influenza vaccine. Conversely, several studies have indicated that the hepatitis B vaccination may be less effective in smokers than in non-smokers [15,16,24]. Furthermore, the results suggest that there may be a statistically significant impact of smoking on antibody response, contingent on the vaccine formulation. In particular, there appears to be a significant association between smoking and antibody response to mRNA vaccines, whereas the association appears to be relatively weak with inactivated vaccines [65,66]. Nevertheless, the impact of active or previous smoking on the immune response to the SARS-CoV-2 vaccine remains inconclusive, particularly with regard to the underlying mechanisms. Linardou et al. proposed the hypothesis that smoking may exert a suppressive effect on the immune system by directly affecting T cells and dendritic cells, which can impede the vaccination response [26]. A recent study, conducted as part of the VASCO project on healthcare workers, demonstrated that smoking impairs the formation of memory cells, which are essential for maintaining vaccine-induced immunity [31]. Additionally, smoking has been observed to elevate monocyte and macrophage levels, which may potentially influence the clearance of antibodies that typically persist for a duration of 3–4 weeks [67].

This review demonstrates the existence of significant limitations in the research on this topic. Firstly, evidence remains relatively limited, particularly in relation to certain vaccine formulations, such as those based on mRNA. Furthermore, data and findings pertaining to variables such as the duration of smoking, the number of cigarettes smoked per day, passive smoking exposure, and vaccine efficacy are either absent or inconsistent across the various types of vaccines. Moreover, while the majority of retrieved studies employed regression analysis to estimate the independent effect of smoking adjusting for well-established predictors of vaccine response, it is also important to consider that the humoral response to vaccines may be influenced by other factors beyond smoking exposure. These include age, comorbidities, the medication history of the vaccinees, and the number of vaccine doses. Consequently, only preliminary conclusions can be drawn at this stage. In light of these considerations and the inherent limitations in the quality of the data and reporting, our findings should be interpreted with caution and further investigations are required on this issue. In particular, longer controlled studies are needed in order to clarify both the smoking effects on the long-term effectiveness of vaccines and the role of smoking cessation in restoring or improving the vaccine-induced immune response. Nevertheless, the present study represents the inaugural attempt to synthesize epidemiological studies on the impact of smoking on post-vaccination antibody titres in a systematic way. The findings of this research will indubitably inform public health policy and practice. However, the examined studies have demonstrated considerable heterogeneity due to the variability in vaccines and types of exposure investigated. In addition, the effect size may be larger in small studies because we retrieved a biased sample of the smaller studies, and it is also possible that the effect size really is larger in smaller studies—perhaps because the smaller studies used different populations or different protocols than the larger ones. Moreover, it is of the utmost importance to consider the inter-individual variability in immune response when interpreting these data. Further research in this field is necessary to corroborate our findings.

## 5. Conclusions

Vaccines represent a crucial instrument for the prevention and management of infectious diseases. Evidence suggests that the vaccine-induced immune response is affected by smoking. This review offers a framework for policymakers to address health disparities between smokers and non-smokers. The implementation of health education programs that encourage smoking cessation may enhance the efficacy of immunization campaigns. However, as of today, policies on smoking cessation have limited effectiveness. Thus, in order to enhance the vaccine-induced immune response in smokers, the opportunity to adopt different vaccine dosing schemes for smokers and non-smokers should be considered, especially in the case of acute epidemics. In this perspective, differences in smoking habits across different countries and cultural contexts should be taken in account.

## Figures and Tables

**Table 1:** Main characteristics and results of the included studies.

Author, Year, Country	Study Design	Sample Size, Type of Vaccine, Study Population, Type of Smoking, Immune Response Indicators	Main Results	Quality/Risk of Bias
MacKenzie et al. [14] 1976 UK	RCT	799 Influenza vaccine Group A: 259 (smokers 42.9%), two doses of a live influenza A virus vaccine administered intra-nasally; Group B: 264, two doses of a saline control; Group C: 276 (smokers 32.2%), two doses of a killed subunit influenza vaccine administered by deep subcutaneous injection Active tobacco cigarette smoking Serum antibody levels	After receiving the live vaccine, smokers seroconverted significantly higher with respect to non-smokers (*p* < 0.05). Subunit vaccines determined a similar trend. After vaccination, the persistence of the immunity was assessed for 50 weeks. The persistence of haemagglutination-inhibiting (HI) antibody was not significantly different between smoker and non-smoker subjects who received the live vaccine, while a significant depression in the persistence of HI antibody by 50 weeks was observed in smokers who received subunit vaccines. The persistence in volunteers with residual immunity before vaccination was not influenced by smoking.	Medium risk
Struve et al. [15] 1992 Sweden	Non-randomized CT	595 Hepatitis B vaccine: 257 intramuscular route, 338 intradermal route Female (74.8%); median age 34–42 years; smokers (39%) Active tobacco cigarette smoking Serum antibody levels	Smokers and non-smokers presented a percentage of protective anti-HBs levels (≥10 IU/I) respectively equal to 39% and 61%. Female sex, intramuscular vaccination, young age, and being a non-smoker were linked to a higher geometric mean anti-HBs titre and a higher response rate with respect to males, intradermal vaccination, old age and being a smoker. The vaccination’s failure rate was equal to an OR 1.6 (95% CI 0.9–2.9, NS) comparing smokers vs. non-smokers.	Medium risk
Winter et al. [16] 1994 UK	RCT	115 Hepatitis B vaccine Group A: 56 (rapid schedule, vaccination at 0, 1, 2 and 12 months); Group B: 59 (standard schedule, vaccination at 0, 1 and 6 months) Female (69.6%); smokers (32.2%); Active tobacco cigarette smoking Serum antibody levels	Regardless of vaccination schedule, smokers (standard vaccination: 28.6%, rapid vaccination: 5.6%) failed to seroconvert and achieve higher antibody levels with respect to non-smokers (standard vaccination: 5.6%, rapid vaccination: 2.8%) (*p* = 0.0003).	Medium risk
Cruijff et al. [17] 1999 Netherlands	Double-blind RCT	1531 Influenza vaccine Group A: 927 Purified split-virion vaccine, A/Singapore/6/86 (H1N1), A/Beijing/353/89 (H3N2), B/Panama/45/90 and B/Beijing/1/87; Group B: 911 intramuscular placebo physiological saline solution Female (52.3%); age range 60–91 years; cigarette smokers (321), pipe and/or cigar smokers (58), non-smokers (1152); 490 patients with heart conditions, lung conditions, or diabetes mellitus Cigarette, pipe and cigar active smoking Serum antibody levels	The efficacy of vaccination was found to be statistically significantly different between smokers and non-smokers (*p* < 0.0001; corrected for age, sex and risk group). No notable discrepancies were observed in pre-titres between smokers and non-smokers, with adjustments made for age, sex, and risk group. The antibody titre exhibited a statistically significant increase following vaccination in smokers for the A/Singapore/6/86 and B/Beijing/11/87 strains, but not for the A/Beijing/353/89 and B/Panama/45/90 strains. The decline in titre at the conclusion of the post-vaccination period was marginally more pronounced in smokers for all strains. The end-titre was observed to be marginally elevated in smoker with respect to non-smoker individuals both for B/Panama/45/90 and B/Beijing/11/87. In addition, the end-titre adjusted for age, sex and risk group was significantly higher for A/Singapore/6/86 (*p* = 0.04).	Low risk
Baynam et al. [18] Australia 2007	Cross-sectional study	200 with parental atopic history Pertussis, polio, haemophilus influenzae type b, measles–mumps-rubella vaccines Female: no parental smoking (39%), parental smoking (55%); mean age 2 years and 15 days; parental smoking: 158 (79%) not exposed, 42 (21%) exposed Passive tobacco cigarette smoking Serum antibody levels	The unexposed subjects with IL-4 2589 CT/TT genotypes exhibited heightened antibody responses to diphtheria (*p* = 0.005) and tetanus (*p* = 0.04) compared with those with the CC genotype. Individuals with IL-4Ra I5OV II/IV genotypes exhibited heightened antibody responses to diphtheria compared with subjects with the VV genotype, though this did not reach statistical significance (NS). Subjects who had been exposed and who carried the IL-4 2589 CT/TT genotypes exhibited diminished antibody responses to tetanus in comparison with subjects with the CC genotype (*p* = 0.02). Individuals with IL-4Ra Q551R QR/RR genotypes exhibited diminished antibody responses relative to subjects with the QQ genotype (diphtheria, *p* = 0.03; tetanus, *p* = 0.02). Increased vaccine response was associated with single nucleotide polymorphisms in the IL-4 and IL-4Ra genes linked to atopy in children of non-smokers and lowered responses were observed in children of smokers.	Good
Roseman et al. [19] 2012 Sweden	Cohort study	505 Pneumococcal conjugate vaccine Female (63%); smokers (17.4%); ex-smokers (40%); 253 rheumatoid arthritis; 252 spondylarthropathy Active tobacco cigarette smoking Serum geometric mean concentration of antibody responses; serum antibody levels	Serotype specific IgG against pneumococcal polysaccharide serotypes 23F and 6B resulted as follows: 9.9 g/L in smokers (*p* = 0.028), 10.6 g/L in non-smokers (NS), 10.4 g/L in ex-smokers (NS). Geometric mean concentration (GMC) of antibody responses were significantly higher in non-smokers for both serotypes. GMC immune response 23F: smokers 2.6 (*p* = 0.028), non-smokers 3.7 (NS), ex-smokers 3.3 (NS); GMC immune response 6B: smokers 1.8 (*p* = 0.0042), non-smokers 2.4 (NS), ex-smokers 2.2 (NS). Current smokers presented lower IgG serum levels than non-smokers independently of ongoing treatment or diagnosis (*p* = 0.028). Lower IgG level was statistically significantly associated with higher numbers of cigarettes smoked daily and number of packs smoked annually (*p* = 0.003 and *p* = 0.006, respectively).	Good
Nath et al. [20] 2014 Australia	Cohort study	34 (20 COPD, 14 healthy) Influenza vaccine Female: COPD (35%), non-COPD (42.9%); mean age: COPD 66 years, non-COPD 54 years; smokers: COPD 45%, non-COPD 0% Active tobacco cigarette smoking Serum antibody levels	Vaccine response (expressed as Ab titres 28 days post-vaccination) was not associated with the habit of smoking.	Fair
Namujju et al. [21] 2014 Finland	Cohort study	216 Human papilloma virus vaccine Female (100%); age range 16–17 years; smokers: 46.6% interventions, 56.6% controls; 103 HPV vaccine (interventions), 113 HAV vaccine (controls) Active tobacco cigarette smoking Serum antibody levels; serum antibody avidity	After month 7 post vaccination women who smoked (cotinine level > 20 ng/mL) presented levels of anti-HPV16/18 antibodies similar to those received for non-smoking women. Low-avidity HPV16/18 IgG antibodies were observed in 16% of the vaccinated women, and active smoking determined a three-fold increased risk (95% CI 1.0–9.3) of low-avidity antibodies. Mean absorbance of anti-HPV16 antibodies was equal to 1.97 (±0.78) among non-smokers and 1.88 (±0.73) among smokers, but differences were not significant. Mean absorbance of anti-HPV18 antibodies was 1.44 (±0.85) among non-smokers and 1.36 (±0.76) among smokers, but also in this case differences were not significant.	Good
Hartal et al. [22] 2015 Israel	Cohort study	617 Hepatitis B vaccine Female (0%); age range 18–20 years; 156 smokers (25.7%): 132 from 1–19/day and 24 heavy smokers >20/day Active tobacco cigarette smoking Serum antibody levels	Heavy smokers had a lower rate of seropositivity at encounter 2 one month after the first booster dose (66.7% vs. 88.4%, RR 0.75, *p* = 0.016). Heavy smokers were 5 times less likely to present detectable antibodies after a single booster dose (OR 0.196, 95% CI 0.060–0.641, *p* = 0.007).	Good
Petras and Olear [23] 2018 Czech Republic/Slovakia Work supported by Biodrug s.r.o. Slovakia	Single blind RCT	200 Tetanus vaccines Female (50%); age range 24–65 years; smokers (20.5%) Active tobacco cigarette smoking Serum geometric mean concentration of antibody responses; serum antibody levels; pre-to post-booster level ratio	Smokers had a significantly reduced seroconversion rate both considering crude (0.47; 95% Cl 0.23–0.96) and adjusted ORs (aORs) (0.11; 95% Cl 0.02–0.69). Only 56% of smokers (95% CI 40–72%) had a fourfold increase in antibodies with respect to 73% of non-smokers (95% CI 65–80%) (*p* = 0.019). Any significant difference in post-booster geometric mean concentrations (GMCs) of tetanus antibody (7.3 in smokers with respect to 8.0 in non-smokers, NS) and pre-to post-booster level ratio, including the 95%CI (6.7 in smokers with respect to 7.8 in non-smokers, NS).	Low risk
Meier and Berger [24] 2020 Switzerland	Cohort study	247 (40 non responders NR, 73 low responders LR, 134 responders R) Hepatitis B vaccine Smokers: NR (57.1%), LR (52.5%) R (30.8%) Active tobacco cigarette smoking Serum antibody levels	Smoking presented a strong association with predicted non-response (*p* = 0.011). Individuals was grouped as follows: non-smoker (0 points), 1–9 cigarettes a day (1 point), and ≥10 cigarettes a day (2 points), resulting in a potential score between 0 and 6. Vaccine responders presented a median score equal to 1 (IQR 0–2), low-responders equal to 2 (IQR 1–3), and non-responders equal to 2.5 (IQR 1–4). Only about 5% of the responders, but 35% of the low−/non-responders, presented a score equal to or higher than 4, while about 85% of the responders had a score equal to or less than 2. In an ROC analysis, a high score predicted non-response with a specificity equal to 85% and a sensitivity equal to 47%.	Good
Gumus et al. [25] 2021 Turkey	Cross-sectional study	94 Inactive SARS-CoV-2 vaccine Female (45.7%); mean age 41.0 ± 7.74 years; smokers (36.2%) Active tobacco cigarette smoking Serum antibody levels	Seropositivities predominant in non-smokers compared with smokers after each dose of vaccine (75–64.1% and 25.0–35.9%, *p* = 0.555 and *p* = 0.999, respectively).	Good
Linardou et al. [26] 2021 Greece	Cohort study	288 (189 cancer patients and 99 healthy controls) BNT162b2 COVID-19 mRNA, mRNA-1273 and AZD1222 vaccines Female (56.9%); age from 18 to >85; 159 smokers (55.2%): 88 current smokers and 71 previous smokers Active tobacco cigarette smoking Serum antibody levels	Significant association between IgG titres and smoking status (*p* = 0.017). Post-hoc analysis revealed that those who had never smoked presented significantly higher antibody titres with respect to current smokers (*p* = 0.006).	Good
Nomura et al. [27] September 2021 Japan	Cohort study	378 BNT162b2 COVID-19 mRNA vaccine Female (67.5%); median age 44 years; smokers (40.7%) Active tobacco cigarette smoking Serum median antibody titres; serum antibody levels	The age-adjusted median antibody titres (interquartile range) were found to be equal to −174 and 90, respectively, in those who had always smoked and those who had never smoked (*p* < 0.0001). In both genders, the age-adjusted median antibody titres were significantly lower in individuals who had always smoked than in those who had never smoked. The age-adjusted median antibody titres in males were −246 and 49, and in females were −140 and 95, respectively. The antibody titres were found to be significantly lower in current smokers than in ex-smokers (*p* = 0.019). The age-adjusted median antibody titres were found to be significantly lower in individuals who had always smoked than in those who had never smoked, with a *p*-value of 0.0007 for males and 0.0023 for females. The age-adjusted median antibody titres between current smokers and never smokers were found to be significantly different (*p* < 0.0001), as were the titres between ex-smokers and those who had never smoked (*p* = 0.0019).	Good
Nomura et al. [28] December 2021 Japan	Cohort study	365 BNT162b2 COVID-19 mRNA vaccine Female (68.5%); mean age 43.9 years; 135 smokers (37%): 90 current smokers and 45 ex-smokers Active tobacco cigarette smoking Serum median antibody titres; serum antibody levels	The age-adjusted median (interquartile range) of antibody titres at six months was found to be −97 (−277 to 184) in those who had always smoked and 56 (−182 to 342) in those who had never (*p* < 0.0001). Antibody titres in current smokers were −205, while ex-smokers exhibited a value of −72. Antibody titres in ex-smokers were found to be significantly lower than in those who had never smoked (*p* = 0.0203), and current smokers exhibited lower titres than those who had never smoked (*p* < 0.0001). The median percentage change in antibody titres from three to six months was 28.4% for those who had always smoked and 30.3% for those who had never smoked (*p* = 0.3051). The median percentage change in antibody titres from three to six months was 31.7% for current smokers and 27.4% for ex-smokers (*p* = 0.3853). The median percentage change in antibody titres from three to six months was 31.7% for ex-smokers and 27.4% for those who had never smoked (*p* = 0.2914). The median percentage change in antibody titres from three to six months was 31.7% for current smokers and 27.4% for those who had never smoked (*p* = 0.8809). Significant differences were observed between males and females with regard to age-adjusted median antibody titres, according to smoking status. However, no significant differences were identified in the median percentage change in antibody titres by smoking status, when stratified by sex. Both the groups of those who had always smoked and those who had never smoked had significant sex differences in the median percentage change in Ab titres. Age-adjusted median Ab titres and percentage changes between sex and smoking were found as follows: individuals who had always smoked—Ab, median (IQR), U/mL male/female −120/−68 (*p* = 0.5709), percentage change, median (IQR) −25.9%/−30.5% (*p* = 0.0400); those who had never smoked—115/46 (*p* = 0.4700), −24.0%/−31.7% (*p* = 0.0050); *p*-value for Ab titre, male *p* = 0.0040, female *p* = 0.0120; *p*-value for percentage change male *p* = 0.7613, female *p* = 0.7018.	Good
Pitzalis et al. [29] 2021 Italy	Cohort study	975 (912 multiple sclerosis patients and 63 healthy controls) BNT162b2 COVID-19 mRNA vaccine Female (73.1%); mean age 48.8 years in multiple sclerosis patients and 52.1 years in controls; smokers (28.6%) Active tobacco cigarette smoking Serum antibody levels	Effects of cigarette smoking on humoral response to SARS-CoV-2 vaccine in a subset of 535 multiple sclerosis patients negative for Anti-N antibody production for whom smoking status was 28.6% for active smokers. Among multiple sclerosis patients, reduced Anti-S antibody production in smokers (median = 719 U/mL) with respect to non-smokers (median = 1054 U/mL) in response to BNT162b2 vaccine (*p* < 0.001).	Good
Tsatsakis et al. [30] 2021 Greece	Cross-sectional study	517 BNT162b2 COVID-19 mRNA vaccine Female (66.3%); mean age 47.7 years; smokers (34.4%) Active tobacco cigarette smoking Serum antibody levels	Significantly higher Ab titres in non-smokers with respect to smokers (4.48 ± 2.79 vs. 3.80 ± 2.64, respectively, *p* = 0.003).	Good
Ferrara et al. [31] 2022 Italy	Cohort study	162 BNT162b2 COVID-19 mRNA vaccine Female (58.0%); mean age 42.4 years; smokers (30.2%) Active tobacco cigarette smoking Serum median antibody titres; serum antibody levels	A median antibody titre of 211.80 AU/mL (interquartile range [IQR] 149.80–465.50) was observed in 30.2% of current smokers and 69.8% of non-smokers (*p* = 0.002) 60 days after the completion of the vaccination cycle. A median antibody titre of 487.50 AU/mL (IQR 308.45–791.65) was observed in 48.7% of current smokers and 51.3% of non-smokers (*p* = 0.002) 60 days after the completion of the vaccination cycle. The notable disparity in vaccine-induced IgG titres between current smokers and non-smokers persisted even after adjusting for age, sex, and previous SARS-CoV-2 infection.	Good
Uysal et al. [32] 2022 Turkey	Cohort study	314 Inactive SARS-CoV-2 vaccine Female (57.6%); median age: female 39 years, male 41 years; smokers (32.6%) Active tobacco cigarette smoking Serum antibody levels	Significantly lower Ab titres were recovered in smokers [1–125 U/mL (40.0%), 126–250 U/mL (24.7%), >250 U/mL (27.5%)] with respect to non-smokers [1–125 U/mL (60.0%), 126–250 U/mL (75.3%), >250 U/mL (72.5%)] (*p* = 0.032).	Good
Watanabe et al. [33] 2022 Italy	Cohort study	86 BNT162b2 COVID-19 mRNA vaccine Female (60.5%); median age 43 years; smokers (31.7%); Caucasian ethnicity; hypertension (15.3%) type 2 diabetes (2.4%), dyslipidaemia (7.1%) Active tobacco cigarette smoking Serum antibody levels	Significantly lower antibody levels were found in smokers with respect to non-smokers [1099 (1350) vs. 1921 (1375), respectively, (*p* = 0.007)].	Good
Golec et al. [34] 2022 Poland	Cohort study	243 BNT162b2 COVID-19 mRNA vaccine Female (76.95%); mean age 47.42 years; smokers (18.52%) Active tobacco cigarette smoking Serum antibody levels	Following vaccination, smokers exhibit diminished long-lasting immunity relative to non-smokers (OR 0.38, 95% CI 0.17–0.85, *p* = 0.02). The temporal changes in IgG titre are contingent upon the status of smoking.	Good
Hikary et al. [35] 2022 Spain	Cross-sectional study	2467 Human papilloma virus vaccine Female (100%); age range 20–24 years; smokers (18.7%) Active tobacco cigarette smoking Incidence of HPV-related lesions	HPV bivalent or quadrivalent vaccination is effective in protecting against CIN but insufficient in smokers. In non-smokers, HPV vaccination significantly reduced the incidence of HSIL+ from 0.42% to 0.1% (OR 0.21, 95% CI, 0.05–0.95), but not in smokers (OR 0.59, 95% CI, 0.22–1.56). In vaccinated women, the incidence of CIN2+ was equal to 0.20% in non-smokers and 0.87% in smokers (OR 0.22, 95% CI, 0.05–0.89, *p* = 0.02). In the vaccinated cohort, the incidence of CIN1+ was found to be equal to 4.8% in smokers and 1.9% in non-smokers, while the incidence of CIN2+ was 0.87% and 0.20%, respectively. The odds ratio (OR) for non-smokers with respect to smokers in the development of CIN1+ was 0.38 (95% CI, 0.22–0.65, *p* = 0.0003), while the OR for CIN2+ was 0.22 (95% CI, 0.05–0.89, *p* = 0.02).	Good
Mori et al. [36] 2022 Japan	Cross-sectional study	55 BNT162b2 COVID-19 mRNA vaccine Female (0%); age range 20–69 years; smokers (100%): cigarette 25.5%, heat-not-burn tobacco 52.7%, combination 21.8% Active tobacco cigarette smoking, heat-not-burn tobacco Serum antibody levels	Fagerstrom test for nicotine dependence showed a significant negative relationship with IgG levels (ρ = −0.426, *p* = 0.001) and a weak negative relationship between serum cotinine level and IgG concentrations (ρ = −0.156, *p* = 0.256).	Good
Ramirez Sevilla et al. [37] 2022 Spain	Cohort study	1003 MV140 vaccine Female (82.7%); mean age 78 years; smokers (24.6%) Active tobacco cigarette smoking Incidence of urinary tract infections	Smoking did not degrade the response of MV140 in preventing recurrent urinary tract infections. Efficacy was respectively equal to 0–1 urinary tract infections (UTIs) in 80.2% (3 months), 65.5% (6 months), 53.9% (12 months) for smokers and 0–1 UTIs in 85.8% (3 months), 66.8% (6 months), 20% (12 months) for non-smokers (NS).	Good
Trontzas et al. [38] 2022 Greece	Cohort study	246 BNT162b2 COVID-19 mRNA, mRNA-1273 and AZD1222 vaccines Group A: 125 patients with lung cancer and ongoing anticancer therapy, female (25.7%), median age 68 years, smokers (16.0%), ex-smokers (80.0%), those who had never smoked (4.0%); Group B: 35 non-lung cancer patients, female (54.3%), median age 59 years, smokers (14.3%), ex-smokers (68.6%), those who had never smoked (17.1%); Group C: 86 healthy controls; female (72.1%), median age 50 years, smokers (29.1%), ex-smokers (22.1%), those who had never smoked (48.8%) Active tobacco cigarette smoking Serum antibody levels	Significant reduction of Ab titres for lung cancer patients who were smokers with respect to those patients with lung cancer who were former smokers or who had never smoked at T2 (*p* = 0.04), T3 (*p* = 0.04) and T4 (*p* < 0.0001); no significant reduction of Ab titres was recovered for healthy patients smokers (NS).	Poor
Toda et al. [39] 2022 Japan	Cohort study	139 (104 haemodialysis patients and 35 controls) BNT162b2 COVID-19 mRNA vaccine Mean age: haemodialysis patients 70.4 years old, controls 40.3 years old; smokers (9.35%) Active tobacco cigarette smoking Serum antibody levels	Smokers presented significantly lower Ab titres compared with non-smokers (*p* = 0.007)	Fair
Yamamoto et al. [40] 2022 Japan	Cross-sectional study	3433 BNT162b2 COVID-19 mRNA vaccine Female (72.0%); median age 41 years; those who had never smoked (82.0%); ex-smokers (11.0%); smokers (6.0%); exclusive HTPs user (2.0%); dual user of HTPs and cigarettes (1.0%); exclusive cigarette smoker (3.0%); hypertension (8.0%); diabetes (2.0%); cancer (1.0%) Active tobacco cigarette smoking, including heated tobacco products (HTPs) Serum antibody levels; serum geometric mean titres	The antibody geometric mean titre (GMT) was observed to be significantly lower in smokers compared with those who had never smoked (101 vs. 96, respectively; ratio of means, 0.85 [95% CI: 0.78–0.93]). Cigarette smokers exhibited significantly lower GMT than those who had never smoked (adjusted GMT: 118 versus 96; ratio of means: 0.81 [95% CI: 0.71–0.92], *p* < 0.01). Furthermore, exclusive HTP users and dual users exhibited a comparable reduction in adjusted GMT (103 and 107, respectively) relative to those who had never smoked (ratio of means 0.87 [95% CI: 0.75–1.02] and 0.90 [95% CI: 0.75–1.07], respectively, NS).	Fair
Asmar et al. [41] 2023 Palestine	Cross-sectional study	172 COVID-19 mRNA and inactive vaccines Female (45.3%); mean age 39.7 years; smokers (39.5%) Active tobacco cigarette smoking Serum antibody levels	Lower levels of vaccine-induced Ab in smokers with respect to non-smokers with a mean of AU/mL (SD) IgG-S equal to 11,219 (12094) and 13,415 (12972), respectively (NS)	Good
Baglioni et al. [42] 2023 Italy	Cohort study	1115 BNT162b2 COVID-19 mRNA vaccine Female (69.1%); mean age 48.1 years; smokers (23.8%) Active tobacco cigarette smoking Serum antibody levels	Mean Ab response was lower in smokers compared with non-smokers, both at 120 days (787 binding antibody units/mL vs. 949 BAU/mL; *p* < 0.001) and at 180 days from the second dose (493 BAU/mL vs. 657 BAU/mL; *p* < 0.001)	Good
Eyupoglu et al. [43] 2023 Turkey	Cohort study	224 (113 vaccinated) Inactive SARS-CoV-2 vaccine Female (56.3%); median age: female 25.0 years, male 27.0 years; smokers (34.8%); 10 with a history of nCoV (−) inactive vaccine (one dose); 103 with a history of nCoV (−) inactive (two doses) Active tobacco cigarette smoking Serum antibody levels	IgG response rate among participants with a history of nCoV (−) inactive vaccine (one dose) (n = 10) was equal to 0.6 (0.3–4.3) in non-smokers and 0.4 (0.1–18.0) in smokers (NS). IgG response rate in participants with history of nCoV (−) inactive vaccine (two doses) (n = 103) was equal to 49.0 (11.5–160.5) in non-smokers and 23.1 (7.4–98.5) in smokers (NS).	Good
Prather et al. [44] 2023 USA	Cohort study	498 BNT162b2 COVID-19 mRNA, mRNA-1273, Ad26.COV2.S vaccines Female (64.3%); mean age 55; smokers (2%) Active tobacco cigarette smoking Serum antibody levels	Non-smokers presented 2.4-fold higher neutralizing Ab than smokers (mean difference = −0.37, CI −0.64 to −0.10; *p* = 0.007).	Fair
Syrimi et al. [45] 2023 Greece	Cohort study	204 (204 at 4-month timepoint and 189 at 9-month timepoint) BNT162b2 COVID-19 mRNA vaccine Female (51.3%); median age 43 years; smokers (31.8%); Caucasian ethnicity; hypertensive on medication (11), dyslipidaemia (17), autoimmunity (7), immunosuppression (4) Active tobacco cigarette smoking Serum antibody levels	Ab levels increased in non-smokers at the first month [mean 159 U/mL (100–195), *p* = 0.009], at 4 months [mean 24 U/mL (13–49), *p* < 0.001], and at 9 months after the second dose [mean 4.78 U/mL (2.84–8.22), *p* < 0.001] with respect to smokers.	Good
Unal et al. [46] 2024 Turkey	Cross-sectional study	329 Inactive SARS-CoV-2 vaccine Female (71.4%); mean age 49.7 ± 13.7 years; smokers (30.3%) Active tobacco cigarette smoking Serum antibody levels	Ab levels (AU/mL) of individuals with a positive Ab response after vaccination were analyzed by two laboratories, Lab A (183 results) and Lab B (39 results). The resuls were as follows: Lab A: non-smokers (583.38 ± 531.32) and smokers (446.44 ± 392.31) (NS); Lab B: non-smokers (61.07 ± 58.92) and smokers (15.24 ± 16.86) (NS).	Good
Fonzo et al. [47] 2024 Italy	Cohort study	2133 Hepatitis B vaccine Non-smokers (85.8%, female 64.2%), smokers (14.2%, female 53.3%); mean age 20.28 ± 0.92 years Active tobacco cigarette smoking Serum antibody levels	Overall, there was a non-significant difference in the percentage of subjects with Ab levels below 10 IU/L between non-smokers and smokers (50.6% vs. 56.6%). Smokers were more likely to have non-protective Ab levels than non-smokers (AOR: 1.291; 95% CI: 1.006–1.657, *p* = 0.0045).	Good

## Data Availability

No new data were created or analysed in this study. Data sharing is not applicable to this article.
